# Non-alcoholic fatty liver disease associated with gallstones in females rather than males: a longitudinal cohort study in Chinese urban population

**DOI:** 10.1186/s12876-014-0213-y

**Published:** 2014-12-13

**Authors:** Jia Liu, Haiyan Lin, Chengqi Zhang, Lu Wang, Shuo Wu, Dongzhi Zhang, Fang Tang, Fuzhong Xue, Yanxun Liu

**Affiliations:** Department of Epidemiology and Biostatistics, School of Public Health, Shandong University, PO Box 100, Jinan, 250012 China; Health Management Center, Shandong Provincial Qianfoshan Hospital, Jinan, 250014 China; Center for Health Management, Provincial Hospital affiliated to Shandong University, Jinan, 250021 China

**Keywords:** Gallstones, Non-alcoholic fatty liver disease (NAFLD), Longitudinal cohort study, Generalized estimated equation (GEE)

## Abstract

**Background:**

Whether non-alcoholic fatty liver disease (NAFLD) is a risk factor for gallstones remains uncertain. Few longitudinal or cohort studies have been used to identify this relationship. The aim of this study was to confirm the association between NAFLD and gallstones in a longitudinal cohort of urban dwellers in China.

**Methods:**

To elucidate the association between NAFLD and gallstones, we fitted a generalized estimating equation (GEE) model in a large-scale longitudinal cohort over 6 years, which included 11,200 participants with at least three regular health check-ups.

**Results:**

A total of 498 cases of gallstones occurred during the 6-year follow-up, which resulted in a total incidence density of 12.73 per 1000 person-years (498/39, 135.5 person-years). The GEE analyses confirmed and clarified the association between NAFLD and gallstones (relative risk (RR) = 1.2381, 95% confidence interval (CI) = 1.003–1.528, *P* = 0.047) after adjusting for other potential confounding factors, especially in females (RR = 1.707, 95% CI = 1.245–2.341, *P* = 0.001).

**Conclusions:**

NAFLD is associated with gallstones in an urban Chinese population from the middle to upper socioeconomic strata. Moreover, this association is more strongly apparent in females than in males. Further cohort studies must be conducted to confirm this association in the general population.

**Electronic supplementary material:**

The online version of this article (doi:10.1186/s12876-014-0213-y) contains supplementary material, which is available to authorized users.

## Background

Gallstones are common digestive disorders that constitute a significant health problem in Western countries [1]. In recent years, their presence has also been increasing in China. For persons older than 20 years, the prevalence of gallstone disease is rapidly increasing to as high as 3.8% in southern China and 6.1% in northern China [2]. Some factors including ethnicity [1,3], genetic susceptibility [4], age, gender, obesity [5], insulin resistance [6], and type 2 diabetes mellitus [7], have been reported to be risk factors for gallstone development. However, the pathogenic mechanisms of gallstone formation have not been fully ascertained. Further explorations of risk factors for gallstone development are needed to aid in the early identification and prediction of gallstones.

Non-alcoholic fatty liver disease (NAFLD) describes a wide extent of liver conditions ranging from simple steatosis to non-alcoholic steatohepatitis and cirrhosis. The prevalence of NAFLD is reported as approximately 15% in Asians [8]. It is not yet clear as to what role NAFLD may play in gallstone formation. Various cross-sectional studies have indicated that NAFLD is associated with gallstones [9-13]. The prevalence of gallstone disease was reported to be higher in NAFLD patients than in the general adult population in Italy [9,10]. NAFLD was found to be an independent risk predictor for gallstone disease in Slovakian patients with metabolic risk factors [11]. The association between fatty liver and gallstones was also found in the Chinese [12] and Japanese [13] populations. However, conclusions from cross-sectional studies remain insufficient to confirm whether NAFLD is an independent risk factor for gallstones. There are three potential hypotheses to explain why gallstones are always accompanied by NAFLD. (1) Given that both gallstones and NAFLD have a high prevalence in the general population, it is likely to be a chance co-occurrence. (2) Gallstones and NAFLD share common risk factors [5-8], including obesity, insulin resistance, and type 2 diabetes mellitus. (3) NAFLD is an independent risk factor for gallstones.

Sustained efforts are presently directed at clarifying the relationship between NAFLD and gallstones, with the goal of identifying high-risk groups and providing clues for exploring the pathogenic mechanisms of gallstone formation. Results from a large population-based case–control study in China have shown that a history of liver conditions, especially liver cirrhosis, is a risk factor for gallstone development [14]. However, conclusions from such a case–control study are still not sufficient to confirm whether NAFLD is an independent risk factor for gallstones. Therefore, we conducted a large-scale longitudinal cohort study, based on health check-ups, in an urban Han Chinese population from middle to upper socioeconomic strata to further confirm the association between NAFLD and gallstones. A simple generalized estimating equation (GEE) model was first used to select potential confounding factors of gallstone development, and the multiple GEE model was further adopted to detect the association between NAFLD and gallstones. The longitudinal design allowed us to use repeated observations of the same set of variables during the follow-up, and the GEE model was adjusted to explore the inherent correlations among the observations.

## Methods

### Study samples

**Figure 1:**
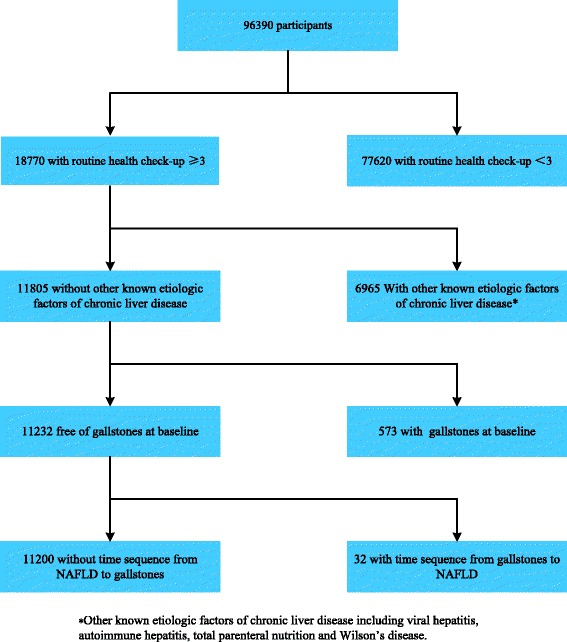
**The longitudinal cohort study sample for analysis of association between non-alcoholic fatty liver disease and gallstones.**

A large-scale longitudinal cohort study was set up in 2005, comprising urban Han Chinese from middle to upper socioeconomic strata who attended a routine health check-up at Centers for Health Management of Shandong Provincial Qianfoshan Hospital and Shandong Provincial Hospital. The geographic area served by the two hospitals covered the city of Jinan, the capital of Shandong province with a population of approximately 7 million. The health check-up database contained relatively rich and educated persons in Jinan, representing the middle to upper class population of Shandong province. Based on the large-scale longitudinal cohort, a sub-cohort was selected from those free of gallstones at baseline. A total of 11,200 participants with at least three health checks in the 6-year follow-up were included in the sub-cohort between 2005 and 2010 (Figure 1).

### Patients’ characteristics and laboratory tests

All participants underwent a general health questionnaire, physical examination, and fasting laboratory assay. The general health questionnaire covered smoking, alcohol intake, sleeping quality, and physical activity. The physical examination included height, weight, systolic blood pressure, and diastolic blood pressure. The body mass index (BMI) was calculated as weight (kg) divided by height (m) squared. After 5 minutes of seated rest, blood pressure was measured from the right arm of the seated participant using an automated sphygmomanometer. Fasting laboratory assays included total glucose, total cholesterol, low-density lipoprotein, high-density lipoprotein, triglycerides, serum total protein, serum albumin, serum globulins, blood urea nitrogen, serum creatinine, hemoglobin, mean corpuscular hemoglobin, red blood cell distribution width, white blood cell count, platelet distribution width, mean platelet volume, and thrombocytocrit. This study was approved by the Ethics Committee of the School of Public Health, Shandong University, and written informed consent for participation in the study was obtained from all participants.

### Definition of gallstones

Ultrasonography was used to diagnose gallstones based on the sonographic evidence of gallstones (one or several echogenic, distally shadowing, possibly movable structures in the gallbladder). Cholecystectomy was defined as the absence of a gallbladder on abdominal ultrasonography. As the individuals in our study were healthy, there were few cases of cholecystectomy after applying exclusion criteria such as fewer than three routine health check-ups. Therefore, cholecystectomy was excluded from our study.

### Definition of non-alcoholic fatty liver disease

According to the revised definition for NAFLD by the Chinese Hepatology Association dating from February 2006 [15], NAFLD was diagnosed by abdominal ultrasonography as brightness in the liver and a diffusely echogenic change in the liver parenchyma, with other known etiologic factors of chronic liver disease (alcohol intake >20 g/day, hepatitis B antigen or hepatitis C virus antibody positive, and autoimmune hepatitis) excluded.

### Missing data imputation

Multiple imputations were used to account for missing values. Since our imputation method was selected depending on the pattern of missing data and the type of the imputed variables, the Markov chain Monte Carlo method was chosen according to the MI Procedure of SAS 9.2 [16]. Data were imputed only for covariates. Before imputation, the extent of missing data in all variables was less than 20%; in particular, less than 17% for drinking, smoking, quality of sleep, physical activity, triglycerides, high-density lipoprotein, and low-density lipoprotein, and 10% for other variables. After imputation, all variables had less than 10% missing observations; in particular, less than 6% for drinking, smoking, quality of sleep, physical activity, triglycerides, high-density lipoprotein, and low-density lipoprotein, and 1% for other variables.

### Statistical analysis

Statistical descriptions are expressed as mean ± standard deviation for continuous variables and as frequencies for categorical variables. Summary statistics were obtained for variables of interest at both baseline and follow-up. Mean values were compared by *t* test for unequal variances. Frequencies were compared by the chi-squared test.

As the age and gender had been confirmed as the chief risk factors for gallstones, the association between gallstones and other factors were examined after adjusting for baseline age; moreover, males and females were analyzed both together and separately. Simple GEE models were first used to select factors associated with gallstones, and multiple GEE models were further adopted to detect the association between NAFLD and gallstones [17]. The GEE models used ‘Logit’ as the link function. Furthermore, the GEE model was fitted to test the regression coefficient of interaction terms between gender and NAFLD status. All statistical analyses were performed using SAS 9.2 (SAS Institute, Cary, NC, USA). A two-sided P value of less than 0.05 was considered to be statistically significant.

## Results

Additional file 1: Figure S1 shows the structure of the cohort during the 6 years of follow-up. Of the 11,200 subjects fulfilling enrollment criteria (5227 males and 5973 females), a total of 498 gallstone events occurred over the 6-year follow-up, leading to a total incidence density of 12.73 per 1000 person-years (498/39, 135.5 person-years), with 7.1, 14.5, 16.6, 18.7, and 2.6 per 1000 person-years of follow-up in the 1st, 2nd, 3rd, 4th, and 5th year, respectively.

The distributions of potential confounding factors of participants grouped by NAFLD status at baseline are shown in Table 1. The baseline prevalence of NAFLD was 30.55% in males and 13.11% in females. At baseline, statistically significant differences between non-NAFLD and NAFLD groups for the listed variables were observed except for red blood cell distribution width, platelet distribution width, and thrombocytocrit in males, and serum creatinine, mean corpuscular hemoglobin, and red blood cell distribution width in females. Additional file 2: Table S1 shows the distributions of potential confounding factors at the baseline survey, and their distributions between subjects with gallstones and subjects without gallstones at each follow-up interval.

**Table 1:** **Distribution of potential confounding factors of participants grouped by NAFLD status at baseline**

	**Male**			**Female**		
	**Non-NAFLD**	**NAFLD**	***P*** **value**	**Non-NAFLD**	**NAFLD**	***P*** **value**
**Sample size**	3630	1597		5308	665	
**Age**	43.14 ± 14.73	51.11 ± 13.89	<0.001	40.65 ± 12.61	54.11 ± 13.29	<0.001
**BMI**	23.47 ± 3.08	27.38 ± 2.82	<0.001	22.83 ± 3.06	27.35 ± 3.23	<0.001
**SBP**	120.81 ± 19.06	135.42 ± 19.49	<0.001	115.94 ± 17.67	136.09 ± 21.31	<0.001
**GLO**	27.44 ± 4.00	28.19 ± 4.30	<0.001	27.81 ± 3.93	29.54 ± 4.41	<0.001
**ALB**	46.35 ± 2.46	46.59 ± 2.40	<0.001	46.02 ± 2.40	45.78 ± 2.56	0.019
**BUN**	4.86 ± 1.26	5.32 ± 1.22	<0.001	4.51 ± 1.16	4.92 ± 1.22	<0.001
**CREA**	77.56 ± 14.39	83.81 ± 14.09	<0.001	70.28 ± 10.12	70.98 ± 10.59	0.096
**GLU**	5.01 ± 0.93	5.56 ± 1.30	<0.001	4.90 ± 0.81	5.73 ± 1.47	<0.001
**CHOL**	4.89 ± 0.94	5.32 ± 0.95	<0.001	4.88 ± 0.97	5.55 ± 1.04	<0.001
**TG**	1.13 ± 0.87	2.06 ± 1.51	<0.001	0.99 ± 0.79	1.91 ± 1.39	<0.001
**HDL-C**	1.38 ± 0.33	1.23 ± 0.31	<0.001	1.47 ± 0.32	1.35 ± 0.32	<0.001
**LDL-C**	2.76 ± 0.73	3.12 ± 0.71	<0.001	2.69 ± 0.74	3.22 ± 0.77	<0.001
**Hemoglobin**	142.43 ± 14.85	151.64 ± 13.22	<0.001	134.20 ± 11.51	138.62 ± 10.96	<0.001
**MCH**	29.85 ± 1.93	29.98 ± 1.70	0.004	29.51 ± 2.10	29.41 ± 2.06	0.275
**RDW-SD**	41.41 ± 2.58	41.49 ± 2.46	0.232	41.35 ± 2.52	41.28 ± 2.30	0.498
**WBC**	6.30 ± 1.50	7.08 ± 1.57	<0.001	6.14 ± 1.47	6.95 ± 1.62	<0.001
**PDW**	12.30 ± 1.72	12.30 ± 1.70	0.977	12.31 ± 1.72	12.16 ± 1.57	0.032
**MPV**	10.41 ± 0.82	10.35 ± 0.80	0.001	10.46 ± 0.81	10.32 ± 0.76	<0.001
**PCT**	0.25 ± 0.08	0.25 ± 0.07	0.797	0.26 ± 0.09	0.27 ± 0.06	<0.001

The abbreviations of the variables: GD = gallstones; BMI = body mass index; SBP = systolic blood pressure; GLO = serum globulins; ALB = serum albumin; BUN = blood urea nitrogen; CREA = serum creatinine; GLU = total glucose; TC = Total cholesterol; TG = triglycerides; LDL = low-density lipoprotein; HDL = high-density lipoprotein; Hb = Hemoglobin; MCH = mean corpuscular hemoglobin; RDW = Red blood cell distribution width; WBC = white blood cell; PDW = Platelet distribution width; MPV = mean platelet volume; PCT = Thrombocytocri.

Table 2 shows the frequency and corresponding event rate per 1000 person-years of gallstone events among subjects with and without NAFLD. Of the 4713 individuals who suffered NAFLD, 289 (6.1%) developed gallstones during the follow-up years, leading to an incidence density of 31.75 per 1000 person-years. For the individuals without NAFLD, 209 of 6487 (3.2%) developed gallstones, with the event rate decreasing to 6.96 per 1000 person-years. When subgroup analysis was performed in males and females, the frequency of gallstones in the NAFLD group was over 20 times higher than in the non-NAFLD group in females, whereas in males the frequency of gallstones in both groups was similar.

**Table 2:** **Gallstone events by non-alcoholic fatty liver disease (NAFLD) status**

		**N (%)**	**Gallstone events (%)**	**Person-year observation**	**Event rate (per 1000 person-years)**
**All**	NAFLD	4713(42%)	289(6.1%)	9103.5	31.7
	non-NAFLD	6487(58%)	209(3.2%)	30032	7
**Male**	NAFLD	2982(57%)	108(3.6%)	6097	17.7
	non-NAFLD	2245(43%)	166(7.4%)	12025.5	13.8
**Female**	NAFLD	1731(29%)	181(10.5%)	3006.5	60.2
	non-NAFLD	4242(71%)	43(1%)	18006.5	2.4

Additional file 3: Tables S2, Additional file 4: Table S3, Additional file 5: Table S4 show the results of age-adjusted simple GEE models in all participants, and in males and females, respectively. In all participants, BMI, systolic blood pressure, serum albumin, serum globulins, glucose, and triglycerides were statistically significant (Additional file 3: Table S2). In gender-specific analyses, BMI, systolic blood pressure, serum albumin, serum globulins, glucose, triglycerides, and white blood cell count were statistically significant for males (Additional file 4: Table S3). Serum albumin, serum globulins, and glucose were statistically significant for females (Additional file 5: Table S4).

Table 3 shows the summarized results of GEE analyses regarding the association between NAFLD and gallstones (for detailed information, see Additional file 3: Tables S2, Additional file 4: Table S3, Additional file 5: Table S4, Additional file 6: Table S5, Additional file 7: Table S6 and Additional file 8: Table S7). The following results were obtained. (1) NAFLD was associated with gallstones (relative risk (RR) = 1.427, *P* < 0.001); after adjusting for age in all participants (Additional file 3: Table S2), this association was still significant (RR = 1.238, 95% confidence interval (CI) =1.003–1.528, *P* = 0.047), after further adjusting for other potential confounders in the multiple GEE model (Additional file 6: Table S5). (2) When using gender-specific analyses the association became stronger in females, not only in the age-adjusted simple GEE model (RR =1.807, 95% CI = 1.277–2.558, *P* = 0.001) but also in the multiple GEE model (RR = 1.707, 95% CI = 1.245–2.341, *P* = 0.001) after further adjusting for other potential confounders (Additional file 4: Tables S3 and Additional file 7: Table S6), whereas no association was detected in males (Additional file 5: Tables S4 and Additional file 8: Table S7). Moreover, the interaction between gender and NAFLD status was statistically significant (*P* = 0.016). These results indicate that NAFLD was an independent risk factor for gallstones in the female rather than the male population.

**Table 3:** **Results of generalized estimating equation (GEE) analysis for non-alcoholic fatty liver disease (NAFLD) and gallstones with their risk ratio (RR) and 95% confidence intervals (CI)**

	**Age-adjusted**			**Multivariate-adjusted**		
	**RR**	**95% CI**	***P*** **value**	**RR**	**95% CI**	***P*** **value**
All	1.427	1.171-1.738	<0.001	1.238	1.003-1.528	0.047
Male	1.188	0.931-1.515	0.166	1.007	0.769-1.318	0.961
Female	1.807	1.277-2.558	0.001	1.707	1.245-2.341	0.001

The association between NAFLD and gallstones was assessed by multiple GEE analysis with age, BMI, SBP, ALB, GLO, TG and GLU adjustment in all participants.

The association between NAFLD and gallstones was assessed by multiple GEE analysis with age, BMI, SBP, GLO, ALB, GLU, TG and WBC adjustment in male.

The association between NAFLD and gallstones was assessed by multiple GEE analysis with gender, age, ALB, GLO and GLU adjustment in female.

## Discussion

In this longitudinal cohort study, our findings have mainly shown that NAFLD is an independent risk factor for gallstone development, especially in females. As the pathogenic mechanisms of gallstone formation have not been fully ascertained, accurate assessment of a high-risk population becomes crucial to decreasing the prevalence of gallstones. Approximately 10% of females with NAFLD developed gallstones during the 6-year follow-up, making this a high-risk population for gallstones.

The relevant findings of this study are as follows. (1) An elevated incidence density of gallstones in the NAFLD group was observed in comparison with the non-NAFLD group. This finding is in accordance with the results of cross-sectional studies in various populations from Italy, Japan, and China [9-14,18]. (2) The time sequence of advancement from NAFLD to the presence of gallstones has been confirmed, providing solid evidence that NAFLD is an independent risk factor for gallstones in females.

Specifically, females with NAFLD are about two times as likely as females without NAFLD to have gallstones, after adjustments for potential confounding factors (see Table 3), but such a trend is not observed in males. Various studies have shown that gallstones are a female-predominant disease [19], whereas NAFLD is predominant in males [20]. However, whether males or females are more susceptible to gallstones in a NAFLD population remains unknown. Although a cross-sectional study showed that the association between NAFLD and gallstones was stronger in males than in females [18], the time sequence between NAFLD and gallstones was not ascertained, owing to the disadvantageous study design. Moreover, this discrepancy might also be due to differences in ethnicity. Our present longitudinal cohort study confirms that NAFLD is associated with gallstones in females rather than males (see Table 3), implying a continuing such trend in the future. Therefore, more attention should be paid to the female population with NAFLD in compiling clinical guidelines. Based on various studies [6,21-27], the authors propose two hypotheses to explain the gender difference regarding the association between NAFLD and gallstones. (1) Males tend to develop an antioxidant reaction through overload of uric acid while females react more easily through glucose metabolism, which is associated with biliary cholesterol secretion and subsequent promotion of cholesterol gallstone formation. (2) Estrogen aggravates lipoapoptosis in females with NAFLD, which results in supersaturated cholesterol of bile, further promoting the formation of crystalline precipitate and ultimately leading to gallstone formation. However, these hypotheses need further research on their molecular mechanisms before they can be verified.

The advantage of the present study is that it is based on a large-scale longitudinal cohort design, which ascertains the time sequence from NAFLD to gallstones to confirm that NAFLD is an independent risk factor for gallstone development. Although the causal relationship between NAFLD and gallstones could not be examined by this longitudinal study, it has nonetheless provided solid evidence that NAFLD is an independent risk factor for gallstones in female subjects. However, some disadvantages do exist in our study. Owing to the inherent weakness of our routine health check-up database, we were unable to obtain information on family history and the use of medication. Considering that the study cohort contained only the urban population of middle to upper socioeconomic strata in Shandong province, and that no information on alanine aminotransferase and aspartate aminotransferase were included, further larger validation cohort studies are needed to confirm our results.

## Conclusion

NAFLD is associated with gallstones in an urban Chinese population of middle to upper socioeconomic strata. In gender-specific analyses, this association was strongly confirmed in females but not in males. Further cohort studies are required to confirm this association in the general population.

## Additional files

Additional file 1: Figure S1. Samples of repeated surveys at each year. Additional file 2: Table S1. Distribution of potential confounding factors. Additional file 3: Table S2. Single-predictor generalized estimating equation (GEE) models in all participants. Additional file 4: Table S3. Single-predictor generalized estimating equation (GEE) models in female. Additional file 5: Table S4. Single-predictor generalized estimating equation (GEE) models in male. Additional file 6: Table S5. Results of multiple generalized estimating equation (GEE) analysis for non-alcoholic fatty liver disease(NAFLD) and gallstones in all participants after adjusting other potential confounding factors. Additional file 7: Table S6. Results of multiple generalized estimating equation (GEE) analysis for non-alcoholic fatty liver disease (NAFLD) and gallstones in female after adjusting other potential confounding factors. Additional file 8: Table S7. Results of multiple generalized estimating equation (GEE) analysis for non-alcoholic fatty liver disease (NAFLD) and gallstones in male after adjusting other potential confounding factors.
