# Supplementary material for: Non-alcoholic fatty liver disease associated with gallstones in females rather than males: a longitudinal cohort study in Chinese urban population
Source: BMC Gastroenterol. 2014 Dec 13;14:213. doi: 10.1186/s12876-014-0213-y (PMC4273434; doi:10.1186/s12876-014-0213-y)
Supplement: Additional file 1: Figure S1. — Samples of repeated surveys at each year. [file 12876_2014_213_MOESM1_ESM.pdf]

	the 1st survey	the 2st survey	the 3st survey	the 4st survey	the 5st survey	the 6st survey
2005	2639	1556 (58.96%)	2259 (85.60%)	1931 (73.17%)	2197 (83.25%)	1093 (41.42%)
2006	5374	4691 (87.29%)	4734 (88.09%)	4477 (83.31%)	3601 (67.01%)	
2007	2278	2169 (95.22%)	2166 (95.08%)	1390 (61.02%)		
2008	909	909 (100.00%)	909 (100.00%)			
total	11200	9325 (83.26%)	10068 (89.89%)	7798 (69.63%)	5798 (51.77%)	1093 (9.76%)
