# Supplementary material for: Non-alcoholic fatty liver disease associated with gallstones in females rather than males: a longitudinal cohort study in Chinese urban population
Source: BMC Gastroenterol. 2014 Dec 13;14:213. doi: 10.1186/s12876-014-0213-y (PMC4273434; doi:10.1186/s12876-014-0213-y)
Supplement: Additional file 2: Table S1. — Distribution of potential confounding factors. [file 12876_2014_213_MOESM2_ESM.docx]

**Table S1 Distribution of potential confounding factors.**

|  |  | baseline | 1 year after baseline | | 2 year after baseline | | 3 year after baseline | | 4 year after baseline | | 5 year after baseline | |
| --- | --- | --- | --- | --- | --- | --- | --- | --- | --- | --- | --- | --- |
| Variable | GD | Mean±Std | N | Mean±Std | N | Mean±Std | N | Mean±Std | N | Mean±Std | N | Mean±Std |
| Age | 0 | 44.75±14.91 | 9190 | 46.14±15.06* | 9811 | 46.74±14.9* | 7536 | 48.97±14.77* | 5642 | 49.84±14.59* | 1076 | 51.28±14.79* |
|  | 1 |  | 73 | 53.06±14.81 | 166 | 57.55±16.16 | 166 | 55.09±15.74 | 156 | 55.85±14.93 | 17 | 62.76±15.4 |
| BMI | 0 | 24.28±3.41 | 9190 | 24.15±3.42 | 9811 | 24.08±3.33* | 7536 | 24.28±3.37* | 5642 | 24.18±3.24* | 1076 | 24.39±3.33* |
|  | 1 |  | 73 | 24.71±3.37 | 166 | 25.18±3.77 | 166 | 25.52±3.63 | 156 | 24.99±3.36 | 17 | 27.13±4.04 |
| SBP | 0 | 123.81±20.04 | 9190 | 122.36±20.02* | 9811 | 122.47±19.75* | 7536 | 123.99±20.37* | 5642 | 124.61±20.32* | 1076 | 124.98±20.88* |
|  | 1 |  | 73 | 128.15±18.81 | 166 | 135.12±21.99 | 166 | 133.54±23.82 | 156 | 131.48±19.17 | 17 | 136.29±23.38 |
| GLO | 0 | 27.59±4.07 | 9190 | 27.68±4.07 | 9811 | 28.75±3.99* | 7536 | 29.66±4.05* | 5642 | 30.89±3.99 | 1076 | 32.04±3.68 |
|  | 1 |  | 73 | 28.05±4.87 | 166 | 29.4±4.18 | 166 | 29.79±4.2 | 156 | 30.61±4.09 | 17 | 33.15±3.15 |
| ALB | 0 | 46.4±2.45 | 9190 | 45.62±2.79 | 9811 | 45.19±2.77* | 7536 | 44.96±2.65 | 5642 | 44.85±2.5 | 1076 | 44.76±2.33 |
|  | 1 |  | 73 | 45.03±2.62 | 166 | 44.36±2.77 | 166 | 44.54±2.66 | 156 | 44.93±2.51 | 17 | 44.5±2.67 |
| BUN | 0 | 4.95±1.27 | 9190 | 4.9±1.26* | 9811 | 4.86±1.29* | 7536 | 4.98±1.3* | 5642 | 5.02±1.28* | 1076 | 5.08±1.33 |
|  | 1 |  | 73 | 5.31±1.59 | 166 | 5.21±1.49 | 166 | 5.31±1.38 | 156 | 5.34±1.44 | 17 | 5.51±2.21 |
| CREA | 0 | 78.84±14.55 | 9190 | 79.4±14.38 | 9811 | 79.53±16.89* | 7536 | 80.28±14.69* | 5642 | 78.92±14.27* | 1076 | 78.93±14.79 |
|  | 1 |  | 73 | 82.36±18.25 | 166 | 83.53±16.79 | 166 | 82.6±13.12 | 156 | 81.44±14.24 | 17 | 83.72±23.15 |
| GLU | 0 | 5.12±1.04 | 9190 | 5.13±1.13 | 9811 | 5.18±1.13* | 7536 | 5.3±1.18* | 5642 | 5.53±1.23 | 1076 | 5.66±1.08 |
|  | 1 |  | 73 | 5.37±1.27 | 166 | 5.6±1.61 | 166 | 5.61±1.42 | 156 | 5.75±1.41 | 17 | 6.05±1.56 |
| TC | 0 | 4.98±0.96 | 9190 | 5.04±0.97 | 9811 | 5.08±0.99* | 7536 | 5.19±1.01 | 5642 | 5.31±1 | 1076 | 5.44±1.05 |
|  | 1 |  | 73 | 5.07±0.88 | 166 | 5.3±1.07 | 166 | 5.2±1 | 156 | 5.44±1.02 | 17 | 5.55±1.33 |
| TG | 0 | 1.32±1.1 | 8513 | 1.38±1.15* | 9610 | 1.36±1.1* | 6958 | 1.4±1.04 | 5484 | 1.46±1.15* | 995 | 1.59±1.38 |
|  | 1 |  | 72 | 1.77±1.13 | 165 | 1.6±1.12 | 158 | 1.54±1.08 | 152 | 1.66±1.01 | 17 | 1.53±0.74 |
| HDL | 0 | 1.35±0.33 | 8513 | 1.32±0.37 | 9610 | 1.31±0.32 | 6958 | 1.36±0.34 | 5484 | 1.43±0.35 | 995 | 1.35±0.33 |
|  | 1 |  | 72 | 1.29±0.37 | 165 | 1.28±0.33 | 158 | 1.33±0.32 | 152 | 1.39±0.35 | 17 | 1.29±0.34 |
| LDL | 0 | 2.84±0.74 | 8513 | 2.88±0.77 | 9610 | 2.88±0.78* | 6958 | 2.9±0.74 | 5484 | 2.97±0.74* | 995 | 3.08±0.75 |
|  | 1 |  | 72 | 2.94±0.69 | 165 | 3.06±0.86 | 158 | 2.97±0.78 | 152 | 3.13±0.8 | 17 | 3.29±1.08 |
| Hb | 0 | 144.32±15 | 9190 | 144.38±15.06 | 9811 | 143.33±15.15 | 7536 | 143.56±15.16 | 5642 | 143.35±15.45 | 1076 | 144.36±15.51 |
|  | 1 |  | 73 | 143.03±17.65 | 166 | 144.18±14.33 | 166 | 144.6±14.05 | 156 | 145.16±14.41 | 17 | 145.94±13.89 |
| MCH | 0 | 29.88±1.89 | 9190 | 30.15±1.98 | 9811 | 30.15±2.08 | 7536 | 30.28±2.13 | 5642 | 30.2±2.15* | 1076 | 30.26±1.98 |
|  | 1 |  | 73 | 30.41±2.19 | 166 | 30.35±1.87 | 166 | 30.47±1.97 | 156 | 30.59±1.71 | 17 | 30.18±1.03 |
| RDW | 0 | 41.43±2.56 | 9190 | 41.23±2.63* | 9811 | 41.21±2.57* | 7536 | 41.28±2.6 | 5642 | 41.25±2.63 | 1076 | 41.04±2.39 |
|  | 1 |  | 73 | 42.07±2.04 | 166 | 41.87±2.76 | 166 | 41.72±2.93 | 156 | 41.64±2.42 | 17 | 41.55±2.42 |
| WBC | 0 | 6.46±1.55 | 9190 | 236.52±53.16 | 9811 | 10.4±0.81* | 7536 | 6.39±1.57 | 5642 | 6.45±1.57 | 1076 | 6.4±1.53 |
|  | 1 |  | 73 | 12.3±1.71 | 166 | 0.25±0.08 | 166 | 6.54±1.53 | 156 | 6.53±1.4 | 17 | 6.9±1.44 |
| PDW | 0 | 12.3±1.71 | 9190 | 12.27±1.71 | 9811 | 12.18±1.65 | 7536 | 12.11±1.64 | 5642 | 12.04±1.6 | 1076 | 11.96±1.65 |
|  | 1 |  | 73 | 12.5±1.78 | 166 | 12.15±1.65 | 166 | 12.08±1.54 | 156 | 12.04±1.58 | 17 | 11.85±1.52 |
| MPV | 0 | 10.4±0.81 | 9190 | 10.4±0.89 | 9811 | 10.38±0.8 | 7536 | 10.35±0.8 | 5642 | 10.39±0.79 | 1076 | 10.39±0.83 |
|  | 1 |  | 73 | 10.49±0.77 | 166 | 10.38±0.81 | 166 | 10.28±0.75 | 156 | 10.35±0.8 | 17 | 10.31±0.89 |
| PCT | 0 | 0.25±0.08 | 9190 | 0.25±0.25 | 9811 | 0.25±0.21* | 7536 | 0.25±0.05 | 5642 | 0.24±0.06 | 1076 | 0.24±0.07 |
|  | 1 |  | 73 | 0.25±0.06 | 166 | 0.24±0.05 | 166 | 0.25±0.05 | 156 | 0.24±0.06 | 17 | 0.24±0.06 |

*P <0.05.

The abbreviations of the variables: GD=gallstones; BMI = body mass index; SBP = systolic blood pressure; GLO = serum globulins; ALB = serum albumin; BUN = blood urea nitrogen; CREA = serum creatinine; GLU = total glucose; TC = Total cholesterol; TG = triglycerides; LDL =low-density lipoprotein; HDL = high-density lipoprotein; Hb = Hemoglobin; MCH = mean corpuscular hemoglobin; RDW = Red blood cell distribution width; WBC = white blood cell; PDW = Platelet distribution width; MPV = mean platelet volume; PCT = Thrombocytocri
