# Supplementary material for: Non-alcoholic fatty liver disease associated with gallstones in females rather than males: a longitudinal cohort study in Chinese urban population
Source: BMC Gastroenterol. 2014 Dec 13;14:213. doi: 10.1186/s12876-014-0213-y (PMC4273434; doi:10.1186/s12876-014-0213-y)
Supplement: Additional file 3: Table S2. — Single-predictor generalized estimating equation (GEE) models in all participants. [file 12876_2014_213_MOESM3_ESM.doc]

**Table S2**

**Single-predictor generalized estimating equation (GEE) models in all participants with their risk ratio (RR) and 95% confidence intervals (CI).**

|  | **Estimate** | **Standard error** | **Z** | **Pr >|Z|** | **RR** | **lower 95 %**  **Confidence Limits** | **upper 95 %**  **Confidence Limits** |
| --- | --- | --- | --- | --- | --- | --- | --- |
| **NAFLD** | **0.3552** | **0.1007** | **3.5274** | **<0.0001** | **1.4265** | **1.1709** | **1.7376** |
| Drinking | 0.0033 | 0.0875 | 0.0378 | 0.97 | 1.0033 | 0.8453 | 1.1909 |
| Smoking | 0.0392 | 0.0352 | 1.1145 | 0.265 | 1.04 | 0.9714 | 1.1142 |
| Sleeping | 0.0149 | 0.0525 | 0.2841 | 0.776 | 1.015 | 0.9159 | 1.125 |
| Exercise | -0.1644 | 0.0933 | -1.763 | 0.078 | 0.8484 | 0.7067 | 1.0186 |
| **BMI** | **0.047** | **0.014** | **3.3612** | **0.001** | **1.0481** | **1.0198** | **1.0773** |
| **SBP** | **0.0075** | **0.0023** | **3.2333** | **0.001** | **1.0075** | **1.003** | **1.0122** |
| **ALB** | **-0.0675** | **0.015** | **-4.5074** | **<0.0001** | **0.9347** | **0.9076** | **0.9626** |
| **GLO** | **0.0446** | **0.0099** | **4.524** | **<0.0001** | **1.0456** | **1.0256** | **1.0661** |
| BUN | 0.0612 | 0.033 | 1.8537 | 0.064 | 1.0631 | 0.9965 | 1.1342 |
| CREA | 0.002 | 0.0024 | 0.8531 | 0.394 | 1.002 | 0.9974 | 1.0067 |
| **GLU** | **0.1179** | **0.0263** | **4.4824** | **<0.0001** | **1.1251** | **1.0685** | **1.1847** |
| TC | 0.0402 | 0.0461 | 0.8726 | 0.383 | 1.0411 | 0.9511 | 1.1395 |
| **TG** | **0.0923** | **0.0249** | **3.7041** | **<0.0001** | **1.0967** | **1.0444** | **1.1517** |
| HDL | 0.0685 | 0.1401 | 0.4894 | 0.625 | 1.0709 | 0.8138 | 1.4094 |
| LDL | 0.0636 | 0.0668 | 0.9534 | 0.34 | 1.0657 | 0.935 | 1.2148 |
| Hb | 2.398 | 0.0032 | 7.5534 | 1 | 11.0012 | 0.9938 | 1.0062 |
| MCH | 0.0498 | 0.0256 | 1.944 | 0.052 | 1.0511 | 0.9996 | 1.1052 |
| SD | 0.0137 | 0.0167 | 0.8185 | 0.413 | 1.0138 | 0.9812 | 1.0475 |
| WBC | 0.0475 | 0.026 | 1.8236 | 0.068 | 1.0486 | 0.9965 | 1.1034 |
| PDW | -0.0062 | 0.0269 | -0.2334 | 0.815 | 0.9938 | 0.9428 | 1.0475 |
| MPV | 0.0026 | 0.0546 | 0.047 | 0.963 | 1.0026 | 0.9012 | 1.1159 |
| PCT | 0.0503 | 0.053 | 0.9491 | 0.343 | 1.0516 | 0.9484 | 1.1666 |

The abbreviations of the variables: Drinking: 0: never, 1: seldom, 2: often, wine, 3: often beer, 4: often, Chinese spirits,5: often, mixed all kinds; Smoking : 0: never,1: seldom ,2: quit,3:1–4/d , 4 : 5 –15/d, 5 : >15/d; Quality of sleep 0: excellent, 1: well, 2: fair 3: poor, 4: very poor (evaluated by themselves); Physical activity 0: never, 1: seldom (1–2 times a week), 2: often or everyday (more than 3 times a week); BMI = body mass index; SBP = systolic blood pressure; GLO = serum globulins; ALB = serum albumin; BUN = blood urea nitrogen; CREA = serum creatinine; GLU = total glucose; TC = Total cholesterol; TG = triglycerides; LDL =low-density lipoprotein; HDL = high-density lipoprotein; Hb = Hemoglobin; MCH = mean corpuscular hemoglobin; RDW = Red blood cell distribution width; WBC = white blood cell; PDW = Platelet distribution width; MPV = mean platelet volume; PCT = Thrombocytocrit.
