# Supplementary material for: Non-alcoholic fatty liver disease associated with gallstones in females rather than males: a longitudinal cohort study in Chinese urban population
Source: BMC Gastroenterol. 2014 Dec 13;14:213. doi: 10.1186/s12876-014-0213-y (PMC4273434; doi:10.1186/s12876-014-0213-y)
Supplement: Additional file 4: Table S3. — Single-predictor generalized estimating equation (GEE) models in female. [file 12876_2014_213_MOESM4_ESM.doc]

**Table S3**

**Single-predictor generalized estimating equation (GEE) models in female with their risk ratio (RR) and 95% confidence intervals (CI).**

|  | **Estimate** | **Standard error** | **Z** | **Pr >|Z|** | **RR** | **lower 95 %**  **Confidence Limits** | **upper 95 %**  **Confidence Limits** |
| --- | --- | --- | --- | --- | --- | --- | --- |
| **NAFLD** | **0.5918** | **0.1773** | **3.3372** | **0.001** | **1.8072** | **1.2766** | **2.5582** |
| drinking | -0.1381 | 0.1332 | -1.0376 | 0.299 | 0.8710 | 0.6709 | 1.1307 |
| smoking | -0.3095 | 0.1126 | -2.7481 | 0.006 | 0.7338 | 0.5886 | 0.9151 |
| sleeping | 0.0332 | 0.0844 | 0.3941 | 0.693 | 1.0338 | 0.8762 | 1.2199 |
| exercise | -0.3147 | 0.1490 | -2.1041 | 0.035 | 0.7300 | 0.5445 | 0.9787 |
| BMI | 0.0209 | 0.0216 | 0.9641 | 0.335 | 1.0211 | 0.9787 | 1.0653 |
| SBP | 0.0061 | 0.0038 | 1.5934 | 0.111 | 1.0061 | 0.9986 | 1.0137 |
| **ALB** | **0.0691** | **0.0149** | **4.6447** | **<0.0001** | **1.0715** | **1.0408** | **1.1034** |
| **GLO** | **-0.0599** | **0.0228** | **-2.6309** | **0.009** | **0.9419** | **0.9008** | **0.9849** |
| BUN | 0.0601 | 0.0490 | 1.2263 | 0.22 | 1.0620 | 0.9647 | 1.1691 |
| CREA | -0.0004 | 0.0054 | -0.083 | 0.934 | 0.9996 | 0.9891 | 1.0101 |
| **GLU** | **0.1479** | **0.0355** | **4.1550** | **<0.0001** | **1.1594** | **1.0812** | **1.2431** |
| TC | 0.1151 | 0.0635 | 1.8125 | 0.07 | 1.1220 | 0.9907 | 1.2707 |
| TG | 0.0604 | 0.0456 | 1.3232 | 0.186 | 1.0622 | 0.9714 | 1.1616 |
| HDL-C | 0.1655 | 0.2094 | 0.7903 | 0.429 | 1.1800 | 0.7828 | 1.7787 |
| LDL-C | 0.1325 | 0.1182 | 1.1211 | 0.262 | 1.1417 | 0.9057 | 1.4394 |
| Hb | -0.0037 | 0.0051 | -0.7375 | 0.461 | 0.9963 | 0.9863 | 1.0063 |
| MCH | 0.0653 | 0.0372 | 1.7568 | 0.079 | 1.0675 | 0.9925 | 1.1481 |
| SD | 0.0078 | 0.0250 | 0.3099 | 0.757 | 1.0078 | 0.9595 | 1.0585 |
| WBC | 0.0003 | 0.0429 | 0.0076 | 0.994 | 1.0003 | 0.9197 | 1.0880 |
| PDW | -0.0246 | 0.0409 | -0.604 | 0.546 | 0.9757 | 0.9005 | 1.0570 |
| MPV | -0.0096 | 0.0798 | -0.1207 | 0.904 | 0.9904 | 0.8470 | 1.1581 |
| PCT | 0.0560 | 0.0425 | 1.3158 | 0.188 | 1.0576 | 0.9734 | 1.1495 |

The abbreviations of the variables: Drinking: 0: never, 1: seldom, 2: often, wine, 3: often beer, 4: often, Chinese spirits,5: often, mixed all kinds; Smoking : 0: never,1: seldom ,2: quit,3:1–4/d , 4 : 5 –15/d, 5 : >15/d; Quality of sleep 0: excellent, 1: well, 2: fair 3: poor, 4: very poor (evaluated by themselves); Physical activity 0: never, 1: seldom (1–2 times a week), 2: often or everyday (more than 3 times a week); BMI = body mass index; SBP = systolic blood pressure; GLO = serum globulins; ALB = serum albumin; BUN = blood urea nitrogen; CREA = serum creatinine; GLU = total glucose; TC = Total cholesterol; TG = triglycerides; LDL =low-density lipoprotein; HDL = high-density lipoprotein; Hb = Hemoglobin; MCH = mean corpuscular hemoglobin; RDW = Red blood cell distribution width; WBC = white blood cell; PDW = Platelet distribution width; MPV = mean platelet volume; PCT = Thrombocytocrit.
