# Supplementary material for: Non-alcoholic fatty liver disease associated with gallstones in females rather than males: a longitudinal cohort study in Chinese urban population
Source: BMC Gastroenterol. 2014 Dec 13;14:213. doi: 10.1186/s12876-014-0213-y (PMC4273434; doi:10.1186/s12876-014-0213-y)
Supplement: Additional file 5: Table S4. — Single-predictor generalized estimating equation (GEE) models in male. [file 12876_2014_213_MOESM5_ESM.doc]

**Table S4**

**Single-predictor generalized estimating equation (GEE) models in male with their risk ratio (RR) and 95% confidence intervals (CI).**

|  | **Estimate** | **Standard error** | **Z** | **Pr >|Z|** | **RR** | **lower 95 %**  **Confidence Limits** | **upper 95 %**  **Confidence Limits** |
| --- | --- | --- | --- | --- | --- | --- | --- |
| NAFLD | 0.1720 | 0.1243 | 1.3835 | 0.1660 | 1.1876 | 0.9309 | 1.5153 |
| drinking | 0.0014 | 0.1263 | 0.01072 | 0.991 | 1.0014 | 0.7818 | 1.2825 |
| smoking | 0.0522 | 0.0365 | 1.43079 | 0.152 | 1.0536 | 0.9809 | 1.1316 |
| sleeping | -0.0007 | 0.0691 | -0.0109 | 0.991 | 0.9993 | 0.8728 | 1.1441 |
| exercise | -0.0838 | 0.1209 | -0.6934 | 0.488 | 0.9196 | 0.7256 | 1.1655 |
| **BMI** | **0.0633** | **0.0192** | **3.2959** | **0.001** | **1.0653** | **1.0260** | **1.1062** |
| **SBP** | **0.0087** | **0.0029** | **3.0089** | **0.003** | **1.0087** | **1.0030** | **1.0144** |
| **ALB** | **0.0270** | **0.0134** | **2.0160** | **0.0440** | **1.0274** | **1.0008** | **1.0547** |
| **GLO** | **-0.0780** | **0.0207** | **-3.7766** | **<0.0001** | **0.9250** | **0.8881** | **0.9632** |
| BUN | 0.0555 | 0.0471 | 1.18013 | 0.238 | 1.0571 | 0.9640 | 1.1593 |
| CREA | 0.0031 | 0.0025 | 1.23852 | 0.216 | 1.0031 | 0.9982 | 1.0081 |
| **GLU** | **0.0979** | **0.0378** | **2.5858** | **0.0100** | **1.1028** | **1.0240** | **1.1877** |
| TC | -0.0445 | 0.0685 | -0.6505 | 0.515 | 0.9565 | 0.8364 | 1.0938 |
| **TG** | **0.0924** | **0.0312** | **2.9580** | **0.003** | **1.0968** | **1.0317** | **1.1661** |
| HDL-C | 0.0231 | 0.1969 | 0.1172 | 0.907 | 1.0233 | 0.6957 | 1.5054 |
| LDL-C | -0.0800 | 0.0943 | -0.8492 | 0.396 | 0.9231 | 0.7673 | 1.1104 |
| Hb | -0.0035 | 0.0061 | -0.5787 | 0.563 | 0.9965 | 0.9848 | 1.0083 |
| MCH | 0.0253 | 0.0392 | 0.6442 | 0.519 | 1.0256 | 0.9497 | 1.1076 |
| SD | 0.0240 | 0.0225 | 1.0710 | 0.284 | 1.0243 | 0.9803 | 1.0704 |
| **WBC** | **0.0737** | **0.0319** | **2.3081** | **0.021** | **1.0765** | **1.0112** | **1.1461** |
| PDW | 0.0026 | 0.0358 | 0.07237 | 0.942 | 1.0026 | 0.9346 | 1.0756 |
| MPV | 0.0079 | 0.0765 | 0.10405 | 0.917 | 1.0079 | 0.8678 | 1.1709 |
| PCT | 0.1337 | 0.9006 | 0.14847 | 0.882 | 1.1431 | 0.1956 | 6.6789 |

The abbreviations of the variables: Drinking: 0: never, 1: seldom, 2: often, wine, 3: often beer, 4: often, Chinese spirits,5: often, mixed all kinds; Smoking : 0: never,1: seldom ,2: quit,3:1–4/d , 4 : 5 –15/d, 5 : >15/d; Quality of sleep 0: excellent, 1: well, 2: fair 3: poor, 4: very poor (evaluated by themselves); Physical activity 0: never, 1: seldom (1–2 times a week), 2: often or everyday (more than 3 times a week); BMI = body mass index; SBP = systolic blood pressure; GLO = serum globulins; ALB = serum albumin; BUN = blood urea nitrogen; CREA = serum creatinine; GLU = total glucose; TC = Total cholesterol; TG = triglycerides; LDL =low-density lipoprotein; HDL = high-density lipoprotein; Hb = Hemoglobin; MCH = mean corpuscular hemoglobin; RDW = Red blood cell distribution width; WBC = white blood cell; PDW = Platelet distribution width; MPV = mean platelet volume; PCT = Thrombocytocrit.
