# Supplementary material for: Non-alcoholic fatty liver disease associated with gallstones in females rather than males: a longitudinal cohort study in Chinese urban population
Source: BMC Gastroenterol. 2014 Dec 13;14:213. doi: 10.1186/s12876-014-0213-y (PMC4273434; doi:10.1186/s12876-014-0213-y)
Supplement: Additional file 6: Table S5. — Results of multiple generalized estimating equation (GEE) analysis for non-alcoholic fatty liver disease(NAFLD) and gallstones in all participants after adjusting other potential confounding factors. [file 12876_2014_213_MOESM6_ESM.doc]

**Table S5**

**Results of multiple generalized estimating equation (GEE) analysis for non-alcoholic fatty liver disease (NAFLD) and gallstones in all participants after adjusting other potential confounding factors with their** **risk ratio (RR) and 95% confidence intervals (CI).**

|  | | **Estimate** | **Standard error** | **Z** | **Pr >|Z|** | **RR** | **lower 95 %**  **Confidence Limits** | **upper 95 %**  **Confidence Limits** |
| --- | --- | --- | --- | --- | --- | --- | --- | --- |
| Intercept |  | -4.6629 | 0.9221 | -5.06 | <0.0001 |  |  |  |
| **age** |  | **0.0248** | **0.0037** | **6.76** | **<0.0001** | **1.0251** | **1.0178** | **1.0325** |
| **NAFLD** | **1** | **0.2136** | **0.1074** | **1.99** | **0.0467** | **1.2381** | **1.0031** | **1.5281** |
| **NAFLD** | **0** | **0** | **0** | **ref** | **ref** | **ref** | **ref** | **ref** |
| BMI |  | 0.0232 | 0.016 | 1.45 | 0.1466 | 1.0235 | 0.9919 | 1.0559 |
| SBP |  | 0.0047 | 0.0024 | 1.93 | 0.0541 | 1.0047 | 0.9999 | 1.0095 |
| **ALB** |  | **-0.0754** | **0.0152** | **-4.95** | **<0.0001** | **0.9274** | **0.9001** | **0.9555** |
| **GLO** |  | **0.0237** | **0.0104** | **2.27** | **0.023** | **1.024** | **1.0033** | **1.0452** |
| TG |  | 0.0518 | 0.0281 | 1.84 | 0.0656 | 1.0532 | 0.9967 | 1.1128 |
| **GLU** |  | **0.0832** | **0.0295** | **2.82** | **0.0048** | **1.0868** | **1.0257** | **1.1515** |

The abbreviations of the variables: BMI = body mass index; SBP = systolic blood pressure; ALB = serum albumin; GLO = serum globulins; TG = triglycerides; GLU = total glucose.
