# Supplementary material for: Non-alcoholic fatty liver disease associated with gallstones in females rather than males: a longitudinal cohort study in Chinese urban population
Source: BMC Gastroenterol. 2014 Dec 13;14:213. doi: 10.1186/s12876-014-0213-y (PMC4273434; doi:10.1186/s12876-014-0213-y)
Supplement: Additional file 7: Table S6. — Results of multiple generalized estimating equation (GEE) analysis for non-alcoholic fatty liver disease (NAFLD) and gallstones in female after adjusting other potential confounding factors. [file 12876_2014_213_MOESM7_ESM.doc]

**Table S6**

**Results of multiple** **generalized estimating equation (GEE) analysis for non-alcoholic fatty liver disease (NAFLD) and gallstones in female after adjusting other potential confounding factors with their risk ratio (RR) and 95% confidence intervals (CI).**

|  | | **Estimate** | **Standard**  **error** | **Z** | **Pr >|Z|** | **RR** | **lower 95 %**  **Confidence Limits** | **upper 95 %**  **Confidence Limits** |
| --- | --- | --- | --- | --- | --- | --- | --- | --- |
| **Intercept** |  | **-4.5621** | **1.1674** | **-3.91** | **<0.0001** |  |  |  |
| **NAFLD** | **1** | **0.5348** | **0.1611** | **3.32** | **0.0009** | **1.7071** | **1.2448** | **2.3408** |
| **NAFLD** | **0** | **0** | **0** | **ref** | **ref** | **ref** | **ref** | **ref** |
| **age** |  | **0.0301** | **0.0051** | **5.89** | **<0.0001** | **1.0306** | **1.0203** | **1.0409** |
| **ALB** |  | **-0.0742** | **0.0213** | **-3.48** | **0.0005** | **0.9285** | **0.8905** | **0.968** |
| **GLO** |  | **0.0427** | **0.0143** | **2.98** | **0.0029** | **1.0436** | **1.0147** | **1.0733** |
| **GLU** |  | **0.0993** | **0.0367** | **2.71** | **0.0068** | **1.1044** | **1.0278** | **1.1867** |

The abbreviations of the variables: GLO = serum globulins; ALB = serum albumin; GLU = total glucose.
