# Supplementary material for: Non-alcoholic fatty liver disease associated with gallstones in females rather than males: a longitudinal cohort study in Chinese urban population
Source: BMC Gastroenterol. 2014 Dec 13;14:213. doi: 10.1186/s12876-014-0213-y (PMC4273434; doi:10.1186/s12876-014-0213-y)
Supplement: Additional file 8: Table S7. — Results of multiple generalized estimating equation (GEE) analysis for non-alcoholic fatty liver disease (NAFLD) and gallstones in male after adjusting other potential confounding factors. [file 12876_2014_213_MOESM8_ESM.doc]

**Table S7**

**Results of multiple generalized estimating equation (GEE) analysis for non-alcoholic fatty liver disease (NAFLD) and gallstones in male after adjusting other potential confounding factors with their risk ratio (RR) and 95% confidence intervals (CI).**

|  | | **Estimate** | **Standard error** | **Z** | **Pr >|Z|** | **RR** | **lower 95 %**  **Confidence Limits** | **upper 95 %**  **Confidence Limits** |
| --- | --- | --- | --- | --- | --- | --- | --- | --- |
| Intercept |  | -2.734 | 1.2863 | -2.13 | 0.0336 |  |  |  |
| NAFLD | 1 | 0.0067 | 0.1372 | 0.05 | 0.961 | 1.0067 | 0.7693 | 1.3175 |
| NAFLD | 0 | 0 | 0 | **ref** | **ref.** | **ref** | **ref** | **ref** |
| BMI |  | 0.0268 | 0.0227 | 1.18 | 0.2379 | 1.0272 | 0.9825 | 1.0739 |
| **SBP** |  | **0.0124** | **0.0027** | **4.55** | **<.0001** | **1.0125** | **1.0071** | **1.018** |
| GLO |  | 0.0139 | 0.0151 | 0.92 | 0.3582 | 1.014 | 0.9843 | 1.0446 |
| **ALB** |  | **-0.1088** | **0.0198** | **-5.5** | **<.0001** | **0.8969** | **0.8628** | **0.9324** |
| **GLU** |  | **0.0939** | **0.0412** | **2.28** | **0.0228** | **1.0984** | **1.0132** | **1.1909** |
| TG |  | 0.0365 | 0.0375 | 0.97 | 0.3303 | 1.0372 | 0.9637 | 1.1162 |
| WBC |  | 0.0266 | 0.0362 | 0.73 | 0.4628 | 1.027 | 0.9566 | 1.1025 |

The abbreviations of the variables: BMI = body mass index; SBP = systolic blood pressure; GLO = serum globulins; ALB = serum albumin; TG = triglycerides; GLU = total glucose; WBC = white blood cell.
